# Rapid expansion of the invasive oyster *Crassostrea gigas* at its northern distribution limit in Europe: Naturally dispersed or introduced?

**DOI:** 10.1371/journal.pone.0177481

**Published:** 2017-05-09

**Authors:** Marc B. Anglès d’Auriac, Eli Rinde, Pia Norling, Sylvie Lapègue, André Staalstrøm, Dag Ø. Hjermann, Jens Thaulow

**Affiliations:** 1 Norwegian Institute of Water Research (NIVA), Oslo, Norway; 2 French Research Institute for Exploitation of the Sea (Ifremer), SG2M-LGPMM, Laboratoire de Génétique et Pathologie des Mollusques Marins, La Tremblade, France; Bigelow Laboratory for Ocean Sciences, UNITED STATES

## Abstract

The Pacific oyster, *Crassostrea gigas*, was introduced to Europe for aquaculture purposes, and has had a rapid and unforeseen northward expansion in northern Europe. The recent dramatic increase in number of *C*. *gigas* populations along the species’ northern distribution limit has questioned the efficiency of Skagerrak as a dispersal barrier for transport and survival of larvae. We investigated the genetic connectivity and possible spreading patterns between Pacific oyster populations on the southern Norwegian coast (4 localities) and Swedish and Danish populations by means of DNA microsatellite analysis of adult oysters, and by simulating larvae drift. In the simulations we used a 3D oceanographic model to explore the influence of recent climate change (1990–2010) on development, survival, and successful spreading of Danish and Swedish Pacific oyster larvae to Norwegian coastal waters. The simulations indicated adequate temperature conditions for development, survival, and settlement of larvae across the Skagerrak in warm years since 2000. However, microsatellite genotyping revealed genetic differences between the Norwegian populations, and between the Norwegian populations and the Swedish and Danish populations, the latter two populations being more similar. This patchwork pattern of genetic dissimilarity among the Norwegian populations points towards multiple local introduction routes rather than the commonly assumed unidirectional entry of larvae drifted from Denmark and Sweden. Alternative origins of introduction and implications for management, such as forecasting and possible mitigation actions, are discussed.

## Introduction

The Pacific oyster, *Crassostrea gigas*, was repeatedly introduced to Europe for aquaculture purposes in the second half of the 20^th^ Century (see [1] for a review), and has established wild populations in the Black Sea, the Mediterranean Sea and along the Atlantic European coasts, to Scandinavia [2]. Temperature conditions north of France were erroneously thought inappropriate for natural reproduction, and the species was actively introduced for aquaculture purposes in the Netherlands [3], Germany [4], Denmark [5], Sweden [6], and Norway [7]. Climate changes and broader eco-physiological tolerances of the species than first supposed [8] are proposed to be the cause of the recently rapid northward expansion of the species from the Wadden Sea to Sweden [3]. From 2007 the species was found in high densities along the Swedish coast [9] and it has been hypothesized that the species has been introduced to Sweden from Denmark through spreading of larvae with coastal currents [10, 11]. In 2005 the species was observed for the first time in the wild on the northern side of the Skagerrak coast, in Norwegian coastal areas [9, 12], presumably due to larva drifted from “parent populations on the Continent” [11]. The repeated introduction of the species to several countries in northern Europe, and multiple documented accounts of its spreading from locations where it has been introduced [13], makes it important to elucidate the processes that cause the observed rapid expansion of the species distribution along its northern distribution limit in Europe. Two mechanisms of introduction have been hypothesized for this region: 1) natural dispersal of larvae across country borders and 2) post-introduction dispersal from local populations founded through other introduction pathways such as i.e. aquaculture, shipping (ballast water, hull fouling), and live trade (live seafood, bait). Moreover, the changing sea temperature conditions within this region [14] may have caused temporal differences in the dispersal abilities of *C*. *gigas* along its northern distribution limit.

**Fig 1:**
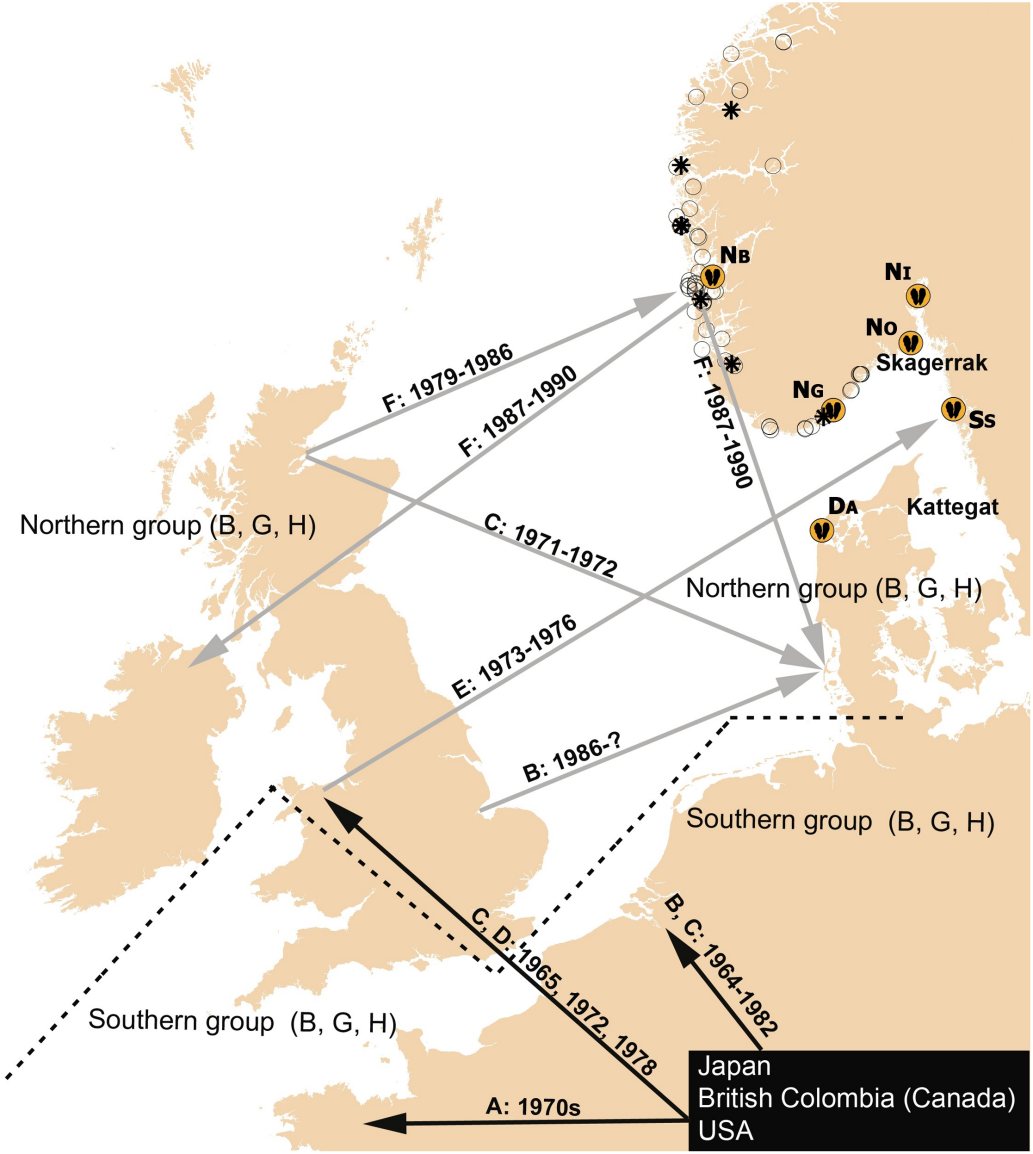
Sampling overview and simplified introduction history. *Crassostrea gigas* aquaculture introduction pathways in Europe (^A^[23], ^B^[17], ^C^[3], ^D^[24], ^E^[9] & ^F^[7]) and the genetic differenciation boundary between a documented southern and northern genetic group delineated by a dotted line (^B^[17], ^G^[15] & ^H^[1]). The six *C*. *gigas* collection sites used in this study are indicated by the oyster symbole (See Table 1 for details). For Norway, valid and withdrawn aquaculture licenses for *Ostrea edulis* (http://www.fiskeridir.no/register/akvareg/?m=utl_lok&s=1; 20. May 2014) and *C*. *gigas* (Directorate of Fisheries) are indicated by open circles and stars, respectively. The map is produced using ESRIs GIS software ArcMap v 10.4.1 (www.esri.com), and the country dataset GISCO NUTS 2010.

*Crassostrea gigas* was introduced to Europe from source populations in either Japan or Canada, which are shown to be genetically similar [1]. However, recent DNA studies of *C*. *gigas* in Europe identify two genetically distinct groups, a northern and a southern. Genetic studies of samples from the south of France to Sweden [15], the south of France to the Wadden Sea [16], samples within the Wadden Sea [17] and samples within the British Isles [1], all indicate two main genetic groups. The two groups seem to be separated by one border in the Wadden Sea and another border within southern UK (Fig 1). The southern group (France, southwestern England, The Netherlands, southern Wadden Sea) with high genetic diversity, was genetically similar to populations from Canada and Japan, whereas the northern group (northern Wadden Sea, Germany, Denmark, Sweden, Ireland and eastern England), with low genetic diversity [1, 15], has, to our knowledge, no genetically matching populations elsewhere in the world. This is consistent with the history of multiple introductions of the species from Canada and Japan to southern Europe, forming a genetically diverse southern group, whereas most of the introductions we are aware of, to the countries belonging to the northern group, come from the UK (see Fig 1 and references). Based on this, the UK appears to be the key source for the Pacific oyster populations within the northern group.

Temperature is a critical factor for *C*. *gigas* larvae development and survival [18]. Maturity and spawning in summer demand temperature above 16–20°C for several days [19, 20]. In warmer water the larvae grow faster [21], the planktonic phase is shorter and a higher proportion of the larvae are successfully metamorphosed [22]. Recent global warming has likely increased the chance of spawning, recruitment, and survival in established populations at the outer edge of its present distribution, accelerating the species proliferation rate and spread to new areas.

Since feral populations of *C*. *gigas* were first observed in Norwegian waters in 2005 [9, 12], the number of known Pacific oyster localities has increased dramatically and the species is at present observed at 435 sites along the Norwegian coastline in Skagerrak and the North Sea (http://artskart.artsdatabanken.no/default.aspx, downloaded 26. February 2017. Some of the 516 observations (81) were duplicates, reported at the same site). This rapid expansion of the species in northern Europe has raised a concern for further uncontrolled northwards expansion through massive larvae supply across Skagerrak from southern countries. This would cause severe problems for any mitigation actions against further northward spread of the species. In this study we used genetic analysis to investigate the origin of 4 established *C*. *gigas* populations along the Norwegian coast. We expect that if the main origin of the Norwegian populations is larvae dispersal from Swedish and Danish populations, then these populations would be genetically similar. Alternatively, if the origin is from post-introduction dispersal from local populations founded through other origins (e.g. aquaculture, shipping, or live trade), we expect these populations to be genetically different. We also examined what influence recent climate change and temperature conditions might have on dispersal of oyster larvae from Swedish and Danish populations, using a 3D oceanographic model, modelled sea water temperature for the region for selected years, and known temperature thresholds for larval development, spawning, and survival.

## Materials and methods

### Sampling and DNA preparation

A total of 262 individuals of *Crassostrea gigas* oysters sampled from six Scandinavian populations in 2010 (Table 1 and Fig 1), were analyzed. As the Pacific oyster is an invasive species considered to be a threat to marine ecosystems in all three countries, permission was not required before sampling. The field studies did not involve endangered or protected species. Shell length varied between 6.5 and 18 cm with an average length of 10.9 cm among sampling locations, implying that all individuals were adults and probably from multiple generations. Oyster mantle samples were collected in 15 ml cap tubes and preserved in ethanol 96% (the ethanol was changed once). We used a new simple DNA preparation protocol without any purification step [25]. Briefly, individual tissue samples were washed in deionized water and about 5 mg mantle tissue was transferred to 100 μl 0.3% SDS with 2 μl proteinase K, incubated at 65°C for 10 min followed by 98°C inactivation for 2 min. The lysates were further diluted 10^−2^ in Tris EDTA buffer (Fluka, Chemie GmbH, Switzerland) prior to performing PCR.

**Table 1:** Sample information.

Country	Region	Code	Date sampled	Sample size	WGS84 DD Lat. / Long.
Norway	Bergen Espevik	N_B_	June 2012	12	59.9159 / 5.64759
Norway	South Norway Grimstad	N_G_	December 2011	50	58.292 / 8.517
Norway	Inner Oslo fjord Sætrepollen	N_I_	May 2012	50	59.68449 / 10.53466
Norway	Outer Oslo fjord Hui	N_O_	May 2012	50	59.11554 / 10.35547
Sweden	Smalsund	S_S_	August 2011	50	58.30262 / 11.36911
Denmark	Agger Tange	D_A_	August 2011	50	56.75923 / 8.24432

Country, region, sample code and size, and geographic position (decimal degrees) of the sampling sites.

### Microsatellite genotyping

PCR amplifications were performed using a CFX96 thermocycler (BioRad, Hercules, CA, USA) in 10 μl reaction volume containing 5 μl iProof mastermix (Bio-Rad), primers (Eurofins MWG, Ebersberg, Germany) were used in two optimized (see Results) multiplex reactions with primer concentrations as indicated in Table 2; Bovine Serum Albumin (BSA) 0.1 μg/μl (VWR, 2 μg/μl) and 2.5 μl sample. Reaction volume was completed with sterile deionised water. Multiplex PCR amplifications were optimized and carried out under the following conditions: a denaturing step for 1 min at 98°C, followed by 35 cycles of 98°C for 15 s, 55°C for 30 s and 72°C for 30 s. Multiplex PCR plates, each with either 4 or 2 different dyes (Table 2), were mixed and diluted by transferring 5 μl from each well to a plate prefilled with 100 μl deionized water per well. From this dilution plate 1.2 μl per sample was transferred to the run plate prefilled with 10 μl HiDi Formamide (Applied Biosystems, Foster City, CA, USA) and 40% strength orange standard (MCLAB, San Francisco, CA, USA). PCR product sizes were determined using a 3730XL DNA analyzer (Applied Biosystems) and scored using GeneMapper software version 4.0 (Applied Biosystems).

**Table 2:** Genotyping of 262 *Crassostrea gigas* individuals using six microsatellite loci in two multiplex PCR.

Locus	Repeat motif	F & R primer sequences 5’-3’	Dye	Conc. (μM)	Size range (bp)	N_A_	N	H_O_	H_E_	Primer Reference
L10*	AG	GGTCAATTCAAAGTCAATTTCCC CATGTTTTCCCTTGACTGATCC	FAM	0.15	109–173	29	262	0.86	0.90	[51]
Cgsili44*	(AG)_7_AAA(GA)_4_ / 25	TGGCATTTCATGGTTAATTT TGTTGTATGAAATGTCGGAA	ATTO 565	0.075	337–363	12	259	0.60	0.83	[52]
HSat1 & HSat2R (AMY)**	TC	ACCGGTATTGCCCGAGTTACAA AGTTAGGCATCCCCCATTGTTC	FAM	0.1	196–238	27	262	0.83	0.89	[53, 54]
L48**	GA	TCAAACCATCTGCTCGTCTACG TCCGAAAATCCAGGAATACCGG	Yakima Yellow	0.2	96–158	26	262	0.86	0.90	[51]
CGE009**	AG	TTCGTTGAAGGTGACAAGTG GCATTTTGGGATGAACAGA	ATTO 565	0.05	102–126	8	262	0.66	0.71	[55]
CG49**	GT	CATCAGGGGTAAATTAAAGTAAGC CCACAGACGATTTCATATATCCTG	ATTO 550	0.05	128–184	26	259	0.60	0.86	[56]

* Duplex PCR reaction ** Fourplex PCR reaction, number of alleles (N_A_), number of individuals that amplified (N), observed heterozygosity (H_O_), expected heterozygosity (H_E_).

### Genetic diversity and structure

Genotyping results were analyzed with Micro-Checker v2.3.3 [26] to identify potential inconsistencies and errors (e.g., null alleles and large allele drop-out). All incidences identified by Micro-Checker were chromatographically inspected before proceeding with further analyses. GenAlEx software v6.5 [27] was used to report overall observed (H_O_) and expected (H_E_) heterozygosity. Genepop v4.2 [28, 29] was used to report observed (H_O_) and expected (H_E_) heterozygosity for each locus within each sampling location. The number of observed alleles (N_A_) and calculated allelic richness (A_R_), compensating for less individuals in N_B_ (Rarefaction option), at each locus within each location was assessed using HP-RARE [30]. Independence among loci was tested by linkage disequilibrium (LD) and Hardy-Weinberg equilibrium (HWE) was calculated to identify loci and populations departing from theoretical equilibrium of allele frequencies, using the Arlequin software v3.5.1.3 [31]. Calculations of statistical significance were corrected for multiple tests according to the B-Y FDR method [32]. A multivariate Discriminant Analysis of Principal Components (DAPC) was used to resolve genetic connectivity between populations through sequential clustering and model selection. The DAPC was performed in R v3.3.2 [33] using the adegenet package [34] and the sampling locations as prior groups. The genetic relationship between samples, at the population level, was evaluated according to Chords distances (*D*_CE_) [35], calculated and bootstrapped 2000 times with MSA v4.05 [36], and presented in a neighbour joining (NJ) tree [37] using the PHYLIP v3.68 software package [38] and SPLITSTREE v4.0 [39]. The significance of splits in the NJ tree were evaluated according to Hillis & Bull [40], i.e., all splits > 70% are considered statistically significant. Calculated pairwise *D*_CE_’s were also used to generate a Principal Coordinates Analysis (PCoA) in GenAlEx v6.5 [27]. Pairwise genetic differentiation was estimated by calculating the fixation index, *F*_ST_ [41], and the statistical significance of the differences between populations was tested by 10,000 permutations of individuals between samples using MSA v4.05.

### Larvae dispersal and survival simulations

Simulation of larvae dispersal and survival to settlement was performed with an open source, numerical 3D oceanographic model (ROMS) [42] with a spatial resolution of 800 m [43]. ROMS has shown accurate results when compared with field observations [44–46] and is a widely used model at both local and global scale (myroms.org). We focused on the influence of recent climate change by performing the simulations for 6 years (1990, 1998, 2002, 2006, 2007 and 2010) representing the climate since the 90s, one cold (2007), two warm (2002 and 2006) and one moderately warm year (2010). For survival, the simulated larvae have to had experienced 225 recruitment degree days [47], and the temperature at the landing site had to be ≥ 18°C (according to Mann, Burreson [48]). Due to the high reproduction capacity (several million larvae per spawning individual), true individual-based modelling was impossible. Hence we used the super-individual approach suggested by Scheffer, Baveco [49], where each modelled individual represents a large number of actual individuals. From each of 44 locations equally distributed along the Danish and Swedish coastline, 7 simulated larvae were released between 1 and 14 August (1 larvae every second day, i.e. 7*44 = 308 larvae per year [50]), and their floating path, experienced degree days and temperature at the landing sites were recorded. This could represent one viable super-individual from each of seven individuals in a small colony on each location. We did not include any behavior or random walk approach, but chose to distribute the time of release within the two first weeks of August, known to be the most relevant period for oyster spawning [12]. The number of landed larvae in Swedish and Norwegian coastal areas was counted within coastal grid cells of 50x50 km resolution for each simulated year, by summing the landed larvae within all the 800 m cells that fall within each of the coarser grid cells.

## Results

### Genetic diversity

A total of eight microsatellite markers [15] were initially tested using a Norwegian oyster sample (N_O_). Two of these microsatellites, CG108 and Cgsili29, failed to amplify and were not further used. The remaining six markers were first tested in simplex PCR to determine optimal annealing temperatures, and thereafter tested in combinations using various primer concentrations and cycling conditions for multiplex PCR testing. Both MgCl_2_ and BSA were also tested as PCR helpers for multiplex optimization. Successful conditions were found for a fourplex and duplex PCR used in this study as described in the methods and Table 2.

Among the six microsatellite loci analysed, four amplified for all samples while CG49 and Cgsili44 each amplified in 259 out of 262 samples (Table 2 and S1 Appendix). All loci were polymorphic and the total number of alleles detected per locus varied from eight to 33 (Table 2 and S1 Appendix). Populations did not differ markedly in allelic richness, with average number of alleles ranging between 7.19, for N_B_ which also had the lowest analysed sample size (n = 12), to10.35 for N_G_ (Tables 1 and 3). The number of private alleles ranged from none at sampling location N_B_ to eight at S_S_ (Table 3). When the four Norwegian sampling locations are compared to a compiled Swedish and Danish sample, private alleles are 25 and 26 respectively. Genetic diversity was homogenous among the populations with the highest expected heterozygosity found in population N_G_ (0.89), the lowest in N_B_ (0.83), and highest and lowest observed heterozygosity in N_B_ (0.76) and N_I_ (0.71) respectively (Table 3), showing that N_B_ conforms closest to HWE.

**Table 3:** Summary statistics of the six genotyped microsatellites loci in the six analysed sampling locations.

Location	Locus						Mean
	L10	Cgsili44	AMY	L48	CGE009	CG49	
**N**_**B**_							
N_A_	7	5	12	7	5	8	7.3
A_R_	6.83	5.00	11.49	6.83	5.00	8.00	7.19
P_A_	0	0	0	0	0	0	0
H_O_	0.92	0.50	0.92	0.92	0.83	0.45	0.76
H_E_	0.81	0.80	0.91	0.83	0.81	0.80	0.83
p	NS	NS	NS	NS	NS	**	
**N**_**G**_							
N_A_	23	8	20	23	7	16	16.2
A_R_	13.36	7.30	11.65	13.70	5.54	10.57	10.35
P_A_	0	0	0	2	0	2	0.67
H_O_	0.80	0.60	0.84	0.90	0.58	0.64	0.73
H_E_	0.95	0.85	0.91	0.95	0.76	0.91	0.89
p	NS	***	NS	NS	**	**	
**N**_**O**_							
N_A_	21	9	16	18	7	15	14.3
A_R_	11.64	7.36	10.67	10.31	5.21	8.79	9.00
P_A_	0	0	0	0	1	0	0.17
H_O_	0.72	0.76	0.78	0.96	0.68	0.58	0.75
H_E_	0.90	0.86	0.88	0.89	0.72	0.86	0.85
p	***	NS	NS	NS	NS	***	
**N**_**I**_							
N_A_	25	9	19	21	6	13	15.5
A_R_	13.52	7.39	10.68	12.51	5.43	9.00	9.76
P_A_	1	0	0	0	0	1	0.33
H_O_	0.98	0.44	0.74	0.82	0.64	0.62	0.71
H_E_	0.95	0.85	0.88	0.93	0.72	0.87	0.87
p	NS	***	*	NS	NS	***	
**S**_**S**_							
N_A_	22	12	24	20	7	18	17.2
A_R_	13.43	7.24	12.68	12.00	5.13	10.16	10.11
P_A_	1	1	3	1	0	2	1.33
H_O_	0.84	0.66	0.90	0.86	0.60	0.56	0.74
H_E_	0.94	0.81	0.93	0.93	0.69	0.89	0.87
p	NS	NS	NS	NS	***	***	
**D**_**A**_							
N_A_	24	11	18	21	7	17	16.3
A_R_	12.89	7.73	10.68	11.65	5.01	9.68	9.61
P_A_	0	0	0	0	0	3	0.50
H_O_	0.92	0.60	0.80	0.72	0.64	0.74	0.74
H_E_	0.94	0.85	0.90	0.92	0.62	0.89	0.86
p	NS	**	NS	*	NS	NS	

Sampling locations (in bold); N_A_—number of alleles, A_R_—allelic richness according to the rarefaction method, P_A_—private alleles, H_O_—observed heterozygosity, H_E_—expected heterozygosity, p—the p-value from the Hardy-Weinberg equilibrium (HWE) test. Hardy-Weinberg after B-Y FDR adjustment (K = 15) significance levels:

* - 5%, ** - 1%, *** - 0.1% and NS -not significant.

Only one pair of loci (CGE009 & CG49) was identified as significantly linked by the LD analysis (B-Y FDR adjusted α = 0.00984). However, since this non-random association among alleles was not consistent among sampling locations and only identified in N_O_, loci CGE009 and CG49 were retained for further analysis. Significant departure from HWE was identified in 13 of the 36 tests (B-Y FDR adjusted α = 0.01198, Table 3). However, no locus showed significant departure from HWE in all sampled locations.

### Population genetic structure

**Fig 2:**
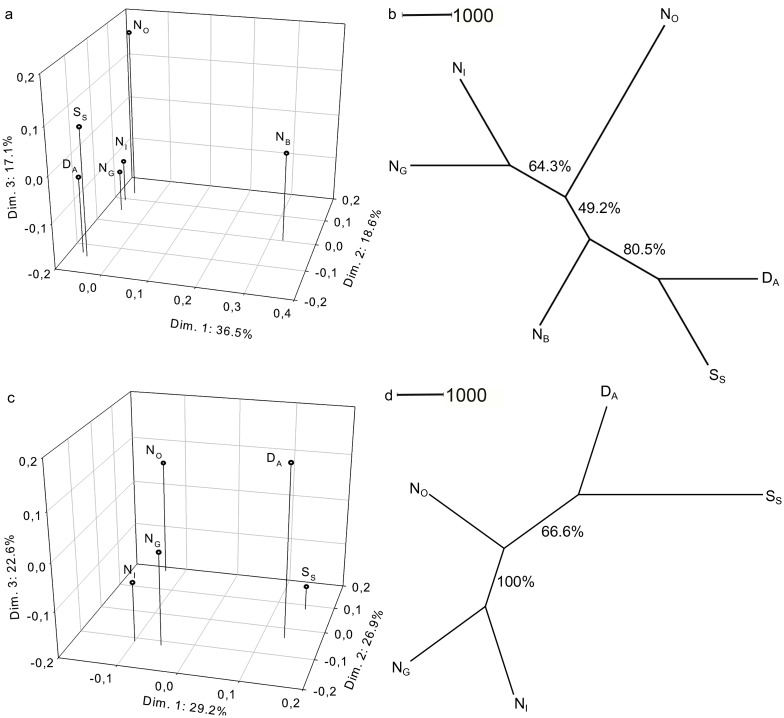
Genetic distance. Similarities and differences among Norwegian (N_B_, N_O_, N_I_, N_G_), Swedish (S_S_) and Danish (D_A_) Pacific oyster (*Crassostrea gigas*) populations visualized by Chords distance [57] in a Principal Coordinate analysis (a and c) and Neighbour Joining tree plot (b and d). Based on all sampled locations (a and b), and for all locations except location N_B_ (c and d), to explore and visualize the genetic distances without location N_B_ that act as an outlier in the data set. Overview of the sampled oyster locations and abbreviations are given in Table 1.

**Fig 3:**
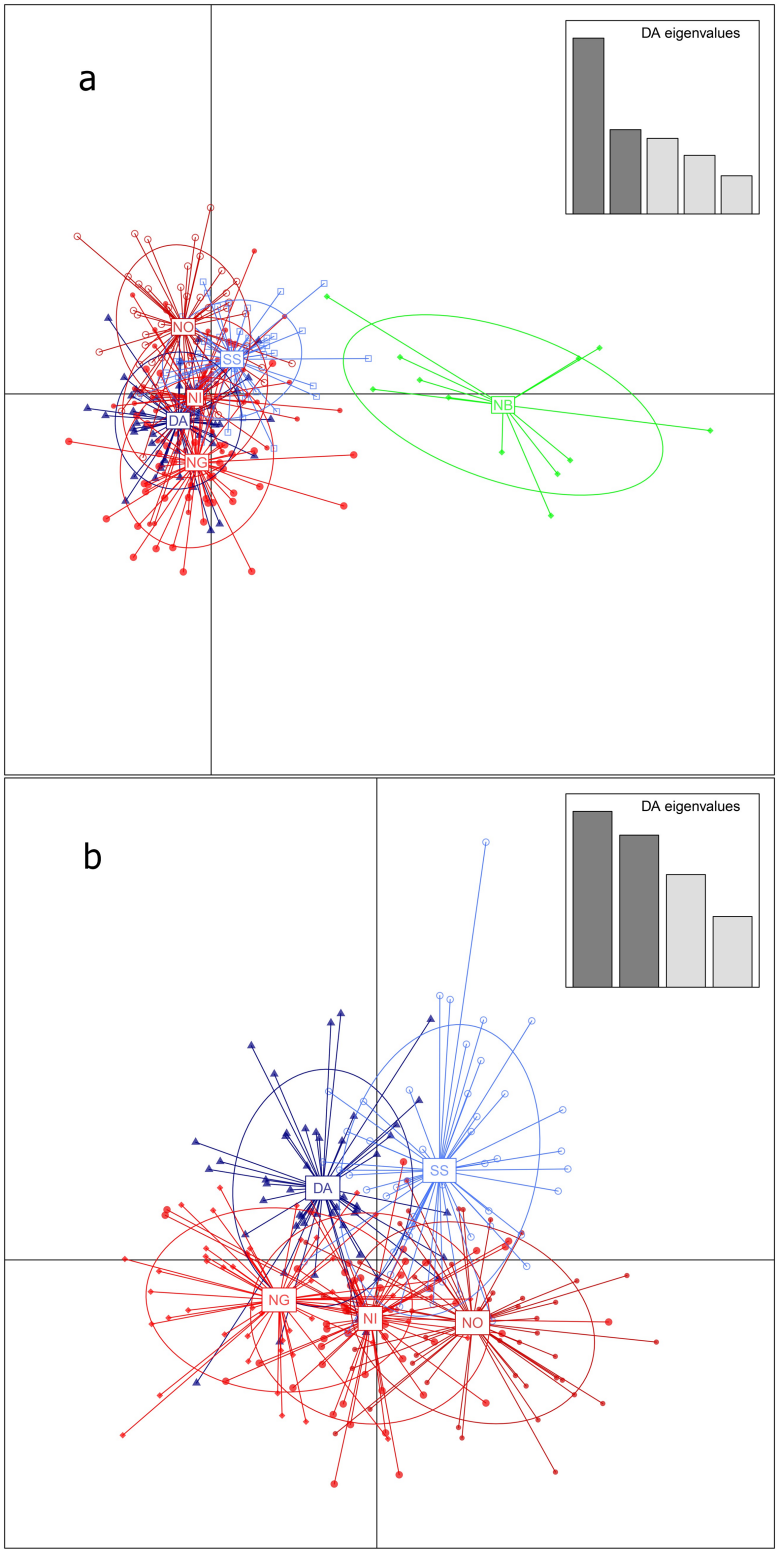
Discriminant Analysis of Principal Components (DAPC). Scatter plot with (a) and without (b) location N_B_ in the analysis. Sampling locations are internally connected with lines to the center of each ellipses. The Danish and Swedish samples are indicated by blue colors (D_A_, dark blue and S_S_, light blue), the Norwegian outlier location (N_B_, green) is differentiated from the remaining Norwegian samples (N_I_, N_O_, and N_G_) represented by red color.

The PCoA analysis of all samples (Fig 2A) separated location N_B_ (at the Norwegian west coast) from the remaining samples along the first dimension representing 36.5% of the total variance (72.2%). The remaining samples clustered into two groups along the second dimension, representing 18.6% of the total variance in the data set. The two clusters were made up of the Swedish (S_S_) and the Danish (D_A_) population in one group and the remaining Norwegian populations (N_I_, N_O_ and N_G_) in the other (Fig 2A). The third dimension separated outer Oslo fjord (N_O_) from the N_I_ and N_G_ samples, representing 17.1% of the variance. The same clustering pattern was also shown by the NJ tree (Fig 2B). In the NJ tree, sampling locations D_A_ and S_S_ were significantly split from the remaining sample locations. The N_B_ sample occurred at an intermediate position within the NJ tree, between the other Norwegian localities (i.e. N_O_, N_I_ and N_G_), and the foreign countries localities (i.e. D_A_ and S_S_, Fig 2B). For both the PCoA and the NJ tree, removal of location N_B_ caused clustering of the remaining samples into three groups: 1) N_O_, 2) N_I_ and N_G_, and 3) S_S_ and D_A_ (Fig 2c and 2d). The DAPC analyses, with all sampling locations included, showed a clear separation of N_B_ from the remaining samples, primarily separated by the first principal component (Fig 3A). The second and third principal components did not vary in information value based on DA eigenvalues. With the removal of N_B_ a stronger tendency for structuring between D_A_ and S_S_ versus the remaining Norwegian samples occurred (N_I_, N_O_, and N_G_, Fig 3B). Despite overlapping of individuals, the population ellipses for the Norwegian samples did not cross the centers of the D_A_ and S_S_ samples. The separation of sampling location N_B_ was supported by high and significant *F*_ST_ values when compared with the remaining sampling locations (Table 4). Among the remaining sampling locations, the pairwise *F*_ST_ values showed non-significant genetic differences, except for the outer Oslo fjord location (N_O_) versus the Danish and Swedish populations (D_A_ and S_S_, respectively, Table 4). Hence all the statistical analyses indicate genetic differentiation.

**Table 4:** Pairwise comparisons of *F*_ST_ (below diagonal) among the six sampled Pacific oyster locations with tested statistical significance between pairs (above diagonal).

	N_B_	D_A_	N_G_	N_I_	S_S_	N_O_
N_B_		***	***	***	***	***
D_A_	0.049		NS	NS	NS	***
N_G_	0.031	0.002		NS	NS	NS
N_I_	0.042	0.006	0.002		NS	NS
S_S_	0.043	0.004	0.004	0.006		***
N_O_	0.046	0.009	0.006	0.004	0.010	

Sample abbreviations are explained in Table 1.

*** - 0.1% and NS -not significant.

### Changes in dispersal and survival of Danish and Swedish oyster larvae

**Fig 4:**
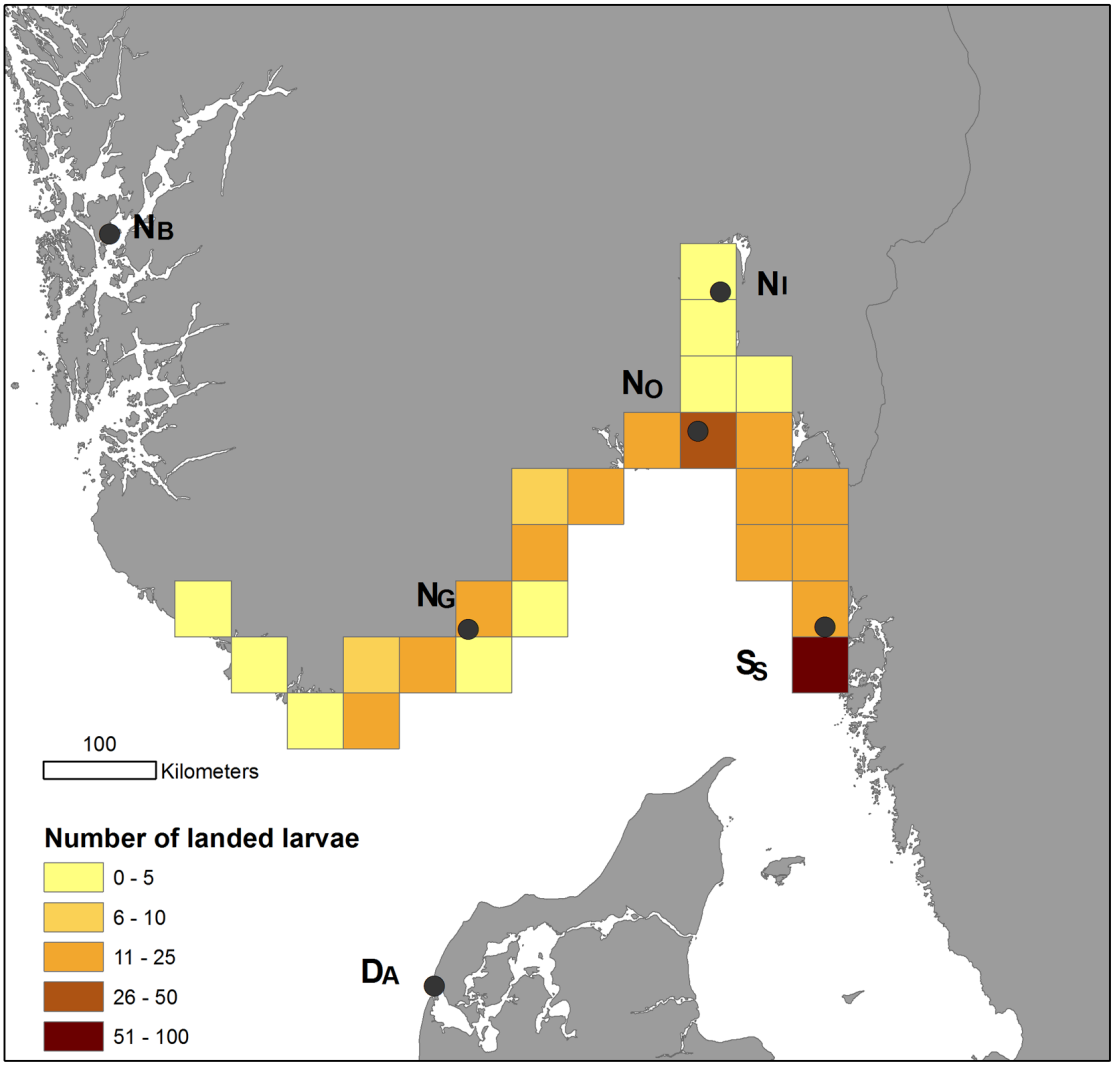
Simulation of larval dispersal. The spatial distribution of the 369 landed Pacific oyster (*Crassostrea gigas*) larvae in Swedish and Norwegian coastal waters in total for the simulated years (1990, 1998, 2002, 2006, 2007, 2010), summed per coastal grid cell (50x50 km). Number of landed larvae (super-individuals) per grid cell is shown (see legend). The location and names of the sampled DNA stations in this study are indicated (black circles, cf. Table 1). For simulation details see [50]. Reprinted from Rinde et al. 2016 under a CC BY license, with permission from NIVA, original copyright 2016. The map is produced using ESRIs GIS software ArcMap v 10.4.1 (www.esri.com), and the country dataset GISCO NUTS 2010.

The simulation results showed that the water temperatures were too cold for the oyster larvae to develop and settle in Norwegian coastal waters in the 1990s (1990 and 1998), but warm summers since 2000 had adequate temperatures for development and survival of transported larvae across the Skagerrak (Table 5 and Fig 4). In the warmest year, 2002, a high fraction (36%) of the released larvae landed in Norwegian coastal waters, whereas in the following cold and moderately warm years (2007 and 2010) only 2–6% of the released larvae experienced sufficient water temperatures to successfully develop and settle on the Norwegian coast. In the two warm years (2002 and 2006), the simulated larvae could reach beyond the limit of the Skagerrak region and into the North Sea region. The hot spot for receiving the highest supply of oyster larvae along the Norwegian coast (i.e. the 50x50 km grid cell with the largest number of landed oyster larvae, Fig 4) included the sampling site at Hui in the Outer Oslo fjord (N_O_).

**Table 5:** Overview of simulated number and fraction of the Pacific oyster (*Crassostrea gigas*) larvae landed in total and within Norwegian coastal waters provided ocean climate in six different years.

Year	1990	1998	2002	2006	2007	2010	In total
*n* released	308	308	308	308	308	308	1848
*n* landed	0	0	192	95	38	44	369
Fraction landed	0	0	0.62	0.31	0.12	0.14	0.20
*n* in Norway	0	0	111	43	7	20	181
Fraction in Norway	0	0	0.36	0.14	0.02	0.06	0.10

## Discussion

The analyses of the Pacific oyster (*Crassostrea gigas*) samples showed that the studied Norwegian populations were genetically different from the Danish and the Swedish populations. This contradicts the hypothesis that the Norwegian populations mainly origin from natural dispersal of larvae drifting from established populations in Denmark and Sweden [11].

Among the studied Norwegian Skagerrak *C*. *gigas* populations, sampling location N_O_ differed genetically from the studied Swedish and Danish populations although it is located within the hot spot area for larvae supply as indicated by the larvae drift simulations. However, the identification of this area as a hotspot area for supply of larvae is otherwise supported by being the only area in Norway with oyster reef formation in 2015 [58]. The genetic difference identified between N_O_ and the Swedish (S_S_) and Danish populations (D_A_) concurs with unpublished genetic studies [10] showing high similarity between Swedish and Danish populations, whereas the population at Hui (N_O_) differed from the studied Swedish and Danish populations. The remaining two Norwegian Skagerrak populations (N_I_ and N_G_), located on each side of N_O_, form a cluster differentiated from both S_S_/D_A_ and N_O_. This patchwork of dissimilar populations across Skagerrak separating the five studied populations into three groups, N_I_/N_G_ and N_O_ on the Norwegian side and S_S_/D_A_ on the opposite side of Skagerrak, is unlikely to be caused by natural dispersal from the S_S_/D_A_-group to Norway. Indeed, genetic discontinuity is observed among three populations along a contiguous stretch of the Norwegian coast (400 km), and only 126 km separates the genetically different populations N_I_ and N_O_. In contrast, the Kattegat Sea separates the Swedish and Danish populations, they are 500 km apart, and yet they form a homogeneous cluster, also different from the three Norwegian Skagerrak populations. Moreover, the larvae drift simulations indicated only two warm years (2002 and 2006) with significant gene flow events across the Skagerrak area since 2000. These two years have the highest summer sea temperatures since measurements on the south coast of Norway (Flødevigen Research station, see www.imr.no) started in 1924. Considering the aquaculture history of *C*. *gigas* in Norway in the 70 – 80s until 2010 [7, 9], when all licenses for Pacific oyster aquaculture in Norway were revoked (Pers. comm. Directorate of Fisheries) it seems more likely that the identified genetic differences separating N_I_/N_G_ from N_O_ and from S_S_/D_A_ are a consequence of multiple introduction events such as from aquaculture or shipping activities. On the other hand, this does not preclude the possible existence of other populations originating from larval drift across the Skagerrak.

The differentiation and independency of the studied Norwegian samples towards the Danish and Swedish samples is furthermore supported by the presence of private alleles in both groups in high and almost equal numbers (25 and 26, respectively). In order for the Norwegian populations to be a result of a frontier/range expansion (drift) scenario, as known from terrestrial [59] and marine organisms [60, 61], the Norwegian Skagerrak group should have had lower genetic diversity, i.e. less private alleles, than the Swedish and Danish populations. Even among the Norwegian populations private alleles occur, providing further evidence of a lack of a uniform population structure, and pointing towards multiple introductions from separate sources of origin.

The larvae drift simulations indicated that the water temperatures in the Skagerrak were too cold for larvae development, survival, and settlement to support successful natural dispersal of Danish and Swedish larva until 2000. Since 2000, summer temperatures have several years been sufficiently high for such natural dispersal and successful crossing of the Skagerrak barrier into Norwegian coastal areas. However, the lack of genetic similarity between the population at Hui (N_O_), which is situated within the hot spot area for landing of foreign *C*. *gigas* larvae in the simulation study, and the studied Danish and Swedish oyster populations, indicates that so far there has been low success rate of this pathway. However, other genetic studies [10] have revealed some similarities between one Norwegian population (approximately 20 km north of N_G_) that was not included in this study, and another Swedish population, which indicates the possible occurrence of successful recruitment of Swedish oyster larvae in Norwegian waters. Future climate change with rising summer temperatures is likely to increase the risk of *C*. *gigas* larvae dispersal [62]. Analysis of sea surface temperature data along the Swedish Skagerrak coast [50] suggests a 125 km northwards displacement of the 19°C temperature isocline in August. This implies a northward shift of the summer temperature needed to enable Pacific oyster spawning in wild populations. This will further push the distribution range of the Pacific oyster northward into previously unfavourable areas/ecosystems, as previously documented for other species [63]. Our findings of a theoretical possible increased supply of foreign *C*. *gigas* larvae in recent and future years clearly indicate a need to monitor and investigate the newly established populations of *C*. *gigas* along the Norwegian coast to assess the connectivity link across the Skagerrak area. There are many factors that may cause high pre- or post-settlement mortality of drifting *C*. *gigas* larva (e.g. predation, starvation, etc.) and that could counteract successful dispersal and colonization across the Skagerrak. In addition, selection imposed by strong environmental gradients, such as the temperature gradient in the studied region, promotes adaptive differentiation [64]. Local adaptation of earlier introduced *C*. *gigas* in Norwegian waters would imply that the local genotypes would have higher fitness than genotypes from foreign habitats [65]. Accordingly, recently landed foreign *C*. *gigas* larvae, would have lower chances of survival than locally adapted oysters.

The simulation model does not include any other mortality factors than the influence of temperature on the larvae’s possibility to develop successfully during the planktonic phase, and sufficient temperature for the larvae to survive at the landing site. This implies that the predicted rate of success of transported Pacific oyster larvae, is likely to be higher than the real success rate. Other mortality factors in the planktonic phase (e.g. starving and predation), when settling (finding suitable substrate), and post settlement (including spatial competition with other species), will all reduce the larvae’s chances of successful spreading. Hence the predicted rate of successful spreading in the two warm years, are likely to be higher than the actual rate because of these limitations, further reducing the possibility of connectivity between the populations.

The genetic differences found among the Norwegian Pacific oyster populations suggests that multiple introductions may have occurred along the coast. This could involve previous aquaculture activities or other introduction pathways such as shipping activities and live trade. Unfortunately, no aquaculture sources were included in the present analysis, so its role as a potential source of introduction cannot be established. Introduction of oysters to ports by shipping is possible since *C*. *gigas* larvae and adults have been found in ballast water and on ship hulls, respectively [66]. The Norwegian Skagerrak coast houses some large ports with high shipping activities and hence the potential for this introduction pathway exists [67]. Spreading of *C*. *gigas* from aquaculture sites has occurred in several countries [13, 68]. In Norway aquaculture licenses for both native (*Ostrea edulis*) and the invasive oyster species (*C*. *gigas*) (Directorate of Fisheries, http://www.fiskeridir.no/register/akvareg/?m=utl_lok&s=1) have been given, indicating that introduction for aquaculture purposes is another plausible introduction pathway. Indeed, the Norwegian population from Bergen (N_B_, western Norway) was collected in the vicinity of a former aquaculture site (Espevik, Tysnes) for *C*. *gigas*, for which the origin of the imported larvae was reported to be Scotland [7]. This population, N_B_, showed strong genetic differentiation from the other studied populations. The possible aquaculture origins for the remaining sampled populations in Norway are difficult to establish from literature. Despite strong restrictions on the import of molluscs for cultivation purposes in Norway in 1986 [9], these aquaculture licenses were still assigned until 2001. The restrictions may have reduced the likelihood of recent repetitive aquaculture introductions.

Although being genetically different, the studied Norwegian oyster populations had low genetic diversity. This agrees with other studies [1, 15, 17] indicating a general pattern of low genetic diversity in the north. Among these studies [15] used the same six microsatellites as this study. Few differences in mean allelic richness were shown for all the analyzed sampling locations (Table 3), except for the westernmost sampling location, Bergen (N_B_). Despite the relatively low number of analyzed individuals, the low allelic richness of the Bergen population could be due to a founder effect or subsequent bottleneck effects [69–71]. However, a bottleneck analysis [72, 73] using the two-phase mutation model with default settings did not identify any bottleneck events in the analyzed samples (data not shown). Moreover, the simulation study indicates low chances for natural dispersal of Danish and Swedish larvae so far along the Norwegian coastline as to Bergen given recent year’s climate. It therefore seems plausible that the Bergen population has adapted to local environmental conditions within the area since the 1970s when the species was introduced for aquaculture purposes. The clustering software STRUCTURE v2.3.4 [74] was run with and without N_B_ (data not shown), to detect any population structure among the sampled populations. The program failed to show any structuring pattern. This concurs with previous *C*. *gigas* studies showing no population structuring except between the northern and the southern European populations [1] or between aquaculture and feral populations [75].

Initial introduction of *C*. *gigas* to Europe entirely originates from Japan, USA, and BC in Canada. Several studies [1, 15, 17] have demonstrated that the southern European populations genetically cluster with the introduction source populations. This indicates that the northern group has developed locally in Europe. As the history of introduction to the north (i.e. to Denmark, Norway and Sweden) mainly originate from the UK following the 1970s, it seems that genetic differentiation between the northern and southern group may have begun in the UK. Hybridization is known to be an evolution mechanism by which genetic variations may be swiftly introduced and fixed in populations, and in particular when species colonize new environments [76]. Considering that *Crassostrea angulata* has been introduced both prior to *C*. *gigas*, and in parallel into the UK [24], hybridization between the two species within the UK may have been possible during this spatial and temporal overlap. Hybridization is documented for *C*. *gigas* with *C*. *angulata* [77, 78], supporting that such an event may have occurred. Although considered as conspecific to *C*. *gigas* by some authors [79], *C*. *angulata* has been shown to be sufficiently genetically distinct to be a separate species [78, 80] and listed under the World Register of Marine Species (http://www.marinespecies.org/aphia.php?p=taxdetails&id=146900, July 2016).

This study contributes to the understanding of the genetic pattern of *C*. *gigas* in northern Europe and shows so far, low connectivity across the Skagerrak. Furthermore, it also demonstrates a likely future increase in successful dispersal of *C*. *gigas* larvae across the Skagerrak, as sea surface temperatures keep rising [14]. The current expansion might therefore temporarily be mitigated by reducing the density of the species in locations with suitable conditions for oyster growth and spawning, e.g. semi-enclosed bays, traditionally used for oyster aquaculture in Norway [7]. The suggested importance of aquaculture, shipping and import for live food as likely introduction pathways is highly relevant for nature management and implies the need to inform aquaculture industries and the public about the risk of introducing invasive species. The future risk of successful dispersal of Pacific oyster larvae from Danish and Swedish populations, due to climate change, emphasize the need for monitoring to detect any massive expansion as basis for targeted management of affected ecosystems. These conclusions may be extended to other invasive species with pelagic larvae stages also affected by climatic change.

## Supporting information

S1 Appendix Genetic data for 262 samples (see Table 1) analyzed with 6 microsatellites (see Tables 2 and 3). (XLSX)
